# The ethical and social significance of NAM terminology and definitions

**DOI:** 10.1016/j.namjnl.2026.100130

**Published:** 2026-09-09

**Authors:** Kevin C. Elliott, Robyn L. Tanguay

**Affiliations:** a Department of Philosophy, Department of Community Sustainability, and Department of Fisheries and Wildlife, Michigan State University, East Lansing, MI, USA; b Sinnhuber Aquatic Research Laboratory, Department of Environmental and Molecular Toxicology at Oregon State University, Corvallis, OR, USA

## Abstract

Definitions for new approach methodologies (NAMs) are highly varied. We argue that current efforts to clarify and harmonize these definitions have paid insufficient attention to their ethical and social significance. Because the choice of definitions influences which methods receive the most policy attention and funding, these definitions are ethically and socially value-laden. To address this issue, we propose three strategies: (1) employing broad definitions that are compatible with multiple values in contexts where the most important values have not been specified; (2) promoting deliberation among stakeholders in an effort to clarify which values should guide future NAM definitions; and (3) aligning NAM definitions with the values of particular initiatives and organizations when their values are clear.

## Introduction

Deciding how to define new approach methodologies (NAMs) is notoriously difficult. In a recent paper aptly titled “Never-ending Acronym Madness,” LaFollette et al. (2026) highlight 14 distinct definitions of NAMs that have been developed in recent years by various regulatory bodies and academic groups. Similarly, Ahluwalia et al. (2026) identify 11 definitions of NAMs and propose a unified definition that focuses on “species-specific methodologies, not including the use of living animals” (p. 3). Macmillan et al. (2026) emphasize that NAM definitions vary in whether or not they include studies involving invertebrates, non-human embryonic vertebrates, animal-derived components, and short-term in vivo studies. They also note that organizations differ in how they define “animals” and “animal-free” and that some organizations have changed their definitions of NAMs over time. Even the acronym ‘NAMs’ is ambiguous, insofar as it sometimes refers to ‘new approach methods’, ‘non-animal methods’, ‘non-animal models’, ‘new alternative methods’, or ‘novel alternative methods’ (Ahluwalia et al., 2026; LaFollette et al., 2026). Some authors also use the acronym ‘NATs’, which refers to ‘Non-Animal Technologies’ (“New Approach Methodologies” (NAMs) and Non-Animal Technologies (NATs)”, 2026). A report from the U.S. National Academy of Sciences made many of these same points and also raised concerns about focusing definitions on what is “new” (because that changes over time) and about focusing on what the definitions exclude (e.g., animal models) rather than on what they include (NASEM, 2023, p. 33).

Although this previous scholarship has been helpful for clarifying different possible definitions, exploring their strengths and weaknesses, and proposing avenues for harmonizing them, the ethical and social significance of NAM definitions has not received adequate attention. Choices involving scientific terminology and definitions can have important ethical and social consequences, such as altering the course of future research, influencing public perceptions, affecting the behavior of decision makers, and altering regulatory outcomes (Elliott, 2009). In this commentary, we highlight the significant economic, animal welfare, and public health implications associated with defining NAMs, and we suggest three avenues for responding to these implications. First, where ethical and social values are not well-specified, definitions for NAMs should be flexible enough to accommodate multiple values rather than foreclosing different perspectives on which ones to prioritize. Second, given the major economic investments and regulatory efforts currently being directed toward NAMs, policy makers should organize broad-based deliberation about how best to define NAMs in light of their ethical and social implications. Third, when initiatives or organizations are organized around particular values, NAM definitions should be aligned with them.

## The ethical and social consequences of scientific terminology and definitions

Literature from the history, philosophy, and sociology of science highlights the ethical and social significance of scientific terms and definitions (Elliott, 2017; Larson, 2011; Ludwig, 2016). For example, there have been extensive debates about the appropriateness of referring to “invasive,” “exotic,” “non-native,” or “alien” species, given the potential for particular terms to import social prejudices into scientific debates and potentially aggravate social debates about immigration (Inglis, 2020; Larson, 2011; Lodge and Shrader-Frechette, 2003). In the medical field, terms like “chronic fatigue syndrome” and “polycystic ovarian syndrome” have been scrutinized for the ways they encourage particular ways of conceptualizing these conditions, which may affect their diagnosis and treatment (Jason et al., 2004; Teede et al., 2026). Terminology can also help to unify fields and draw attention to phenomena, such as when the term ‘endocrine disruption’ galvanized attention around an array of important environmental effects that were not previously conceptualized together (Elliott, 2009; Krimsky, 2003). The phenomenon of endocrine disruption also illustrates the social importance of definitions. Because endocrine disruptors are regulated more stringently than other chemicals in some jurisdictions, debates over how to define endocrine disruptors have been particularly fraught (Michel, 2019; Zoeller et al., 2014). Similarly, the definition of wetlands has been highly controversial at times because of the special protections often granted to lands that receive this designation (Schiappa, 1996).

We argue that choices involving terminology and definitions for NAMs are similarly ethically and socially significant. Increased development and use of NAMs can be defended based on different values, such as lessening animal suffering, eliminating animal use altogether, promoting environmental and public health, increasing economic and social efficiency, or improving mechanistic understanding of chemical activity (see e.g., Macmillan et al., 2026).^1^ Importantly, different definitions for NAMs tend to be more supportive of different values, and in this sense they can be regarded as ethically and socially “value-laden” (Ward, 2021). For example, a NAM definition that excluded all use of animals would likely shift scientific research and development in this direction, which would support the value of eliminating animal research and potentially support the development of more species-specific, human-relevant tests. However, this definition could discourage research on emerging techniques that generate valuable data but involve limited animal use (e.g., daphnia, fish embryos, or short-term in vivo studies). As a result, it could inadvertently limit environmental and public health advances enabled by these approaches. Similarly, if the primary value underlying the pursuit of NAMs were the desire to increase mechanistic understanding of chemical activity, it would neither be necessary nor advisable to exclude all animal use from the definition, because our understanding of whole animal biological function remains incomplete. Admittedly, emerging techniques that make limited use of animals could still be incorporated in integrated assessments and tiered testing frameworks even if they were not counted as NAMs. However, if funding and policy support is strongly directed toward promoting NAMs in the future (as seems to be the case at the U.S. Environmental Protection Agency and National Institutes of Health),^2^ then narrow definitions might limit the range of techniques that receive this support, thereby potentially inhibiting values associated with public health and mechanistic understanding.

From this perspective, recent high-profile definitions of NAMs are value-laden in ethically and socially significant ways. For example, the unified definition proposed by Ahluwalia et al. (2026) explicitly excludes the use of living animals. Similarly, the U.S. National Institutes of Health has initiated a major research program, Complement Animal Research in Experimentation (Complement-ARIE), to advance the development and use of NAMs. It relies on the definition provided in the FDA Modernization Act 2.0, which defines NAMs in terms of in silico, in chemico, and in vitro techniques (Sunderic et al., 2025), thereby excluding in vivo techniques. LaFollette et al. indicate that this accords with most other definitions, which “do not include *in vivo* tests,” although in vivo tests are “debatably tied in by references to the 3Rs” in many definitions (2026, 2). The reason they potentially incorporate in vivo tests is that the 3Rs refer to replacement, reduction, and refinement; therefore, if definitions include approaches that facilitate the 3Rs, they could include test methods that do not completely replace animal studies but that reduce the number of animals needed or lessen their suffering. Nevertheless, the 3Rs have recently been interpreted in a manner that places NAMs squarely in the category of replacement, which would accord with NAM definitions that exclude in vivo tests (Lauwereyns et al., 2024). Interestingly, the European Commission’s recent “Roadmap for Phasing out Animal Testing for Chemical Safety Testing” specifically states that it avoids referring to NAMs because “many definitions exist and some of them still include the use of live animals” (EU Commission, 2026, 4).

## Recommendations

Given that NAM definitions are not neutral but rather promote particular ethical and social values, steps should be taken to thoughtfully manage these definitions. Here, we suggest three measures that could contribute to handling these definitions in a responsible fashion: (1) in the near term, employing definitions that provide flexibility to serve multiple social values; (2) in the mid- to long-term, organizing venues for deliberation among stakeholders about which ethical and social values should guide NAM definitions; and (3) where specific initiatives or organizations prioritize particular values, aligning NAM definitions with those values.

First, considering the diverse values that can justify the pursuit of NAMs, it is generally advisable to employ definitions that do not prematurely exclude the pursuit of some relevant values. From this perspective, choosing definitions that refer to the 3Rs (including not only replacement but also reduction and refinement) is arguably preferable to choosing definitions that exclude the use of animals altogether. Many members of society favor the reduction of animal suffering, but public views on this topic are highly heterogeneous and complex (Bearth et al., 2024; Hagelin et al., 2003; Masterton et al., 2014). Therefore, the desire to exclude the use of animals altogether (including, for example, studies with fish, vertebrate embryos, or invertebrates) is likely to be more narrowly held. Employing NAM definitions that include a wide range of test methods, including those that lessen animal suffering without excluding the use of animals entirely, better allows for these multiple values to be served. An even more inclusive approach would be to employ definitions that incorporate not only the 3Rs but also methods that generate toxicity information more quickly or more accurately. For example, the definition provided by ECHA (2016) included “some ‘conventional’ methods that aim to improve understanding of toxic effects, either through improving toxicokinetic or toxicodynamic knowledge for substances” (p. 6). This definition would serve not only the values of those who seek to promote animal welfare but also the values of those who seek to use NAMs to foster better human risk assessments or more efficient drug development.

Admittedly, the strategy of employing definitions that exclude as few values as possible has limitations. By “casting the net wide,” these definitions may promote a wide array of values while nevertheless lessening the single-minded focus on some of those values. For example, definitions of NAMs that include conventional methods are likely to encourage research on a wide array of test methods, including some that include animals, which could detract from the goal of eliminating animal testing as quickly as possible. Policy makers can help to address this challenge by fostering deliberation among stakeholders about which values should be primary in future research and policy making about NAMs. A wide array of venues is available for promoting this sort of deliberation, including consensus conferences, citizen juries, science courts, surveys, focus groups, advisory committees, and open comment procedures (see e.g., Rowe and Frewer, 2000; Sant’Ambrogio and Staszewski, 2025; Shirk et al., 2012). It is important to recognize that the values that emerge from these deliberations could vary significantly internationally. In some jurisdictions, the complete elimination of animal use might be the primary value, as the EU Roadmap for Phasing Out Animal Testing suggests. In other jurisdictions, a much wider array of goals might emerge from public deliberation about the pursuit of NAMs. In some jurisdictions, there might be highly polarized perspectives about the values that should underpin the pursuit of NAMs, in which case it might be best to maintain inclusive definitions or to employ different definitions in different initiatives or organization.

When specific initiatives or organizations focus on particular values, care should be taken to ensure that they employ definitions that accord with those values. This approach is exemplified by the decision to avoid referring to NAMs in the EU Roadmap because the developers of the Roadmap concluded that some NAM definitions were not aligned with their value of phasing out animal testing entirely. The leaders of other initiatives should similarly reflect on which definitions best serve their values. For example, according to a prominent overview of the COMPLEMENT-Arie initiative, it appears to have multiple important aims: “to 1) better model human health and disease differences in outcomes across populations, 2) develop NAMs that can provide insights into specific biological processes or disease states, 3) validate mature NAMs to support regulatory use and standardization, and 4) complement traditional animal models to make research more efficient and effective” (Sunderic et al., 2025, 1). Given these multiple aims, which are not focused primarily on eliminating the use of animals, this initiative would appear to be well-served by an inclusive definition that allows for the use of a wide array of test methods that could potentially help to achieve these values. Or, if initiatives like this one employ definitions that exclude the use of animals, such as the recent unified definition provided by Ahluwalia et al. (2026), it would be advisable to justify the assumption that animal-free models are best for achieving the aims of modeling human health and disease, providing insight into disease states, and so on.

## Conclusion

We have argued that NAM definitions are not merely technical choices. Because these definitions have the potential to influence which test methods receive attention and funding from policy makers and publics, they are value-laden in ethically and socially significant ways. Therefore, it is important to manage these choices as responsibly as possible. We have argued for three management strategies. First, in the absence of clear guidance about which ethical and social values to promote, a short-term step is to employ definitions that provide flexibility to serve multiple values. Second, over the longer term, policy makers should organize venues for deliberation among stakeholders about which social and ethical values should guide NAM definitions. Third, when specific initiatives or organizations prioritize particular values, care should be taken to align NAM definitions with them.

A potential challenge associated with aligning NAM definitions with the values associated with particular jurisdictions, initiatives, or organizations is that it could detract from efforts to harmonize and standardize definitions internationally (see e.g., LaFollette et al., 2026). This is a legitimate concern because harmonization of definitions is valuable for promoting effective communication and collaboration. Nevertheless, these benefits need to be weighed against the danger of prematurely arriving at harmonized definitions that implicitly privilege the values of some initiatives and organizations over the values of others. Perhaps a highly inclusive harmonized definition could be chosen, but we have noted that this could detract from the efforts of some stakeholders to prioritize very specific values, such as the complete elimination of animal use. A potential compromise would be to develop a highly inclusive definition that also explicitly includes major sub-categories of NAMs, such as those that are species-specific, those that eliminate the use of vertebrate animals, or those that completely eliminate animal use. This could facilitate harmonization and effective communication while also allowing stakeholders with specific priorities to focus on sub-categories of NAMs that accord with their values. Stakeholder deliberation of the kind that we have proposed may also help to generate greater international agreement on the most important values that should underlie a single harmonized NAM definition. We acknowledge the importance of pursuing harmonization, but we call for these efforts to grapple not only with technical questions but also with the ethical and social implications of NAM definitions and the diverse values motivating the pursuit of NAMs.

## CRediT authorship contribution statement

**Kevin C. Elliott:** Writing – review & editing, Writing – original draft, Funding acquisition, Conceptualization. **Robyn L. Tanguay:** Writing – review & editing, Funding acquisition, Conceptualization.

## Declaration of competing interest

The authors declare the following financial interests/personal relationships which may be considered as potential competing interests:

Kevin C. Elliott reports a relationship with 10.13039/100008663Health and Environmental Sciences Institute that includes: travel reimbursement. If there are other authors, they declare that they have no known competing financial interests or personal relationships that could have appeared to influence the work reported in this paper.

## Data Availability

No data was used for the research described in the article.

## Footnotes

1 We refer to these “reasons” as values, which we define broadly as things that are desirable or worthy of pursuit. For further discussion of the different ways in which values can be defined, see Elliott and Korf (2024).

2 See https://www.epa.gov/newsreleases/trump-epa-takes-new-action-eliminate-animal-testing and https://grants.nih.gov/news-events/nih-extramural-nexus-news/2025/07/nih-funding-announcements-to-align-with-nih-initiative-to-prioritize-human-based-research, both accessed on 8/23/26. In this context, an example of the significance of NAM definitions is that some of the EPA’s language focuses on eliminating *mammalian* animal testing and some of it refers to eliminating animal testing in general, which could have a significant impact on whether the EPA supports the future development of NAMs that incorporate non-mammalian animal models.
